# Global research trends and focus on biomarkers in lung cancer immunotherapy: a comprehensive bibliometric insight and visualization analysis (2001-2025)

**DOI:** 10.3389/fimmu.2026.1622573

**Published:** 2026-02-03

**Authors:** Jiangbo He, Chaoyuan Liu, Fang Ma, Yiguang Zhou, Xianling Liu

**Affiliations:** 1 Department of Oncology, The Second Xiangya Hospital, Central South University, Changsha, Hunan, China; 2 Department of Oncology, Guilin Hospital of the Second Xiangya Hospital, Central South University, Guilin, China

**Keywords:** bibliometric, biomarker, genomics, gut microbiome, immune infiltration, immunotherapy, lung cancer

## Abstract

**Background:**

Immunotherapy has revolutionized the therapeutic paradigm for lung cancer, yet its clinical efficacy exhibits significant interpatient heterogeneity. The implementation of personalized immunotherapy critically depends on the precise identification of predictive biomarkers. However, this field is currently characterized by rapid expansion of research outputs alongside marked fragmentation of knowledge domains, while a systematic evaluation of scientific advancements and emerging frontiers remains conspicuously absent. This study aims to synthesize global research achievements to methodologically delineate the evolutionary trajectory of immunotherapy biomarkers in lung cancer, identify research hotspots, and forecast developmental trends.

**Methods:**

A bibliometric analysis was conducted on 6,180 publications (2001–2025) from the Web of Science Core Collection. Advanced tools (Bibliometrix, VOSviewer, CiteSpace) were employed to analyze publication trends, country/institutional contributions, journal influence, author networks, keyword evolution, and citation dynamics.

**Results:**

The field progressed through incubation (2003–2014), rapid expansion (2015–2022), and maturation (2023–2025) phases, with a 28.87% annual growth rate. China led in productivity (9,394 publications), while the U.S. dominated academic impact (93,888 citations). Harvard University Harvard University emerged as the predominant contributor (532 publications). WANG Y was ranked first in the top 10 most prolific authors (97 publications) while RAMALINGAM SS (5,033 citations) was the most cited authors. CANCERS (319 papers; Q1) was the most published journal, while JOURNAL FOR IMMUNOTHERAPY OF CANCER (8,511 citations; Q1) was the most cited journal. Emerging frontiers encompassed genomics, gut microbiome, soluble PD-L1, and immune infiltration patterns, driven by multi-omics integration and artificial intelligence.

**Conclusion:**

This study represents the first bibliometric analysis of biomarkers in lung cancer immunotherapy. Through scientometric visualization tools, our analysis delineates a paradigm shift from reductionist biomarker discovery to multidimensional integration and translational validation. Prospective advancements necessitate leveraging technological innovations, fostering international collaborative networks, and promoting multi-omics convergence. These efforts aim to facilitate a critical transition from population-based therapeutic strategies to precision-driven stratification, ultimately optimizing clinical outcomes and survival benefits for heterogeneous patient populations.

## 1 Introduction

Lung cancer remains the leading cause of global cancer incidence and mortality. According to the latest Cancer Statistics 2024 (1) report released by the American Cancer Society, global new lung cancer cases are projected to reach 2.481 million in 2025, with approximately 1.817 million deaths anticipated. These figures account for over 18% of all cancer-related mortality worldwide. Immunotherapy, particularly anti-PD-1/PD-L1 therapies utilizing immune checkpoint inhibitors (ICIs), has demonstrated promising efficacy in improving overall survival (OS). This therapeutic approach has demonstrated encouraging improvements in OS for a subset of patients, regardless of treatment-line setting or histological classification of lung cancer. Immunotherapy, either as monotherapy or in combination with chemotherapy, has emerged as the standard treatment for non-small cell lung cancer (NSCLC) patients without driver gene mutations. Recent 5-year update data from the CameL-sq trial (2) demonstrated a 27.8% overall survival rate with camrelizumab plus chemotherapy in advanced squamous NSCLC, indicating potential long-term remission in a subset of patients. For extensive-stage small cell lung cancer (SCLC), the IMpower133 study (3) established the survival benefit of combining atezolizumab with chemotherapy, showing a median overall survival of 12.3 months compared to 10.3 months with chemotherapy alone. In early-stage resectable NSCLC, neoadjuvant chemoimmunotherapy has become a clinical standard based on improved pathological complete response (pCR) rates and event-free survival. The CheckMate-816 trial (4) revealed a pCR rate of 24% with nivolumab-chemotherapy combination, correlating with enhanced 5-year survival rates reaching 65% in postoperative patients. However, the treatment response to immunotherapy exhibits considerable variability across patients. This heterogeneity in treatment response has motivated ongoing research efforts to identify more precise biomarkers, aiming to develop personalized therapeutic strategies.

PD-L1 expression is the first widely used biomarker for immunotherapy in lung cancer (5). However, its standalone predictive value has been constrained by dynamic expression patterns and intratumoral spatial heterogeneity (6). While tumor mutational burden (TMB) shows clinical utility, its predictive thresholds vary across cancer types, and standardization of detection methodologies requires further refinement (7). Although MSI-H/dMMR represents the first FDA-approved pan-cancer immunotherapy biomarker, its clinical application in lung cancer is limited by exceptionally low incidence rates, typically below 1% (8). Recent advancements have identified promising novel biomarkers, including 1) dual immune checkpoint targets such as LAG-3 (9) and TIGIT (10); 2) inflammatory and metabolic indicators like neutrophil-to-lymphocyte ratio (NLR) (11) and systemic immune-inflammation index (SII) (12); 3) liquid biopsy markers such as circulating tumor DNA (ctDNA) (13); and 4) microbiome signatures (14). This synergistic optimization of conventional biomarkers with emerging discoveries continues to advance the frontier of precision immunotherapy in lung cancer. However, biomarker research in lung cancer immunotherapy faces substantial challenges throughout its exploratory process, while current clinical applications remain constrained by limitations.

Research on biomarkers in lung cancer immunotherapy has grown rapidly, yet remains fragmented. Although individual studies continue to proliferate, a synthesized overview of the field’s intellectual structure, historical evolution, and emerging frontiers is still lacking. This fragmentation obscures key knowledge gaps, hinders efficient resource allocation, and may delay the translation of biomarker discoveries into clinical practice. Therefore, a systematic, quantitative, and visual synthesis of the entire research landscape is urgently needed to consolidate scattered knowledge, delineate clear developmental trajectories, and pinpoint underexplored areas that warrant future investigation. Bibliometric analysis offers a suitable methodological approach, enabling large-scale data mining, network visualization, and detection of research trends. To our knowledge, no such comprehensive bibliometric assessment has been conducted in this specific domain.

To address this critical need for consolidation and clarity, this study aims to conduct the first comprehensive bibliometric analysis to map the global research landscape and evolutionary trajectory of biomarkers in lung cancer immunotherapy from 2001 to 2025. Through quantitative and visual analytics, we seek to delineate the field’s knowledge structure, identify core research themes and frontier shifts, and provide an evidence-based perspective on future directions to inform strategies for advancing precision immunotherapy in lung cancer.

## 2 Materials and methods

### 2.1 Database and systematic search strategy

**Figure 1:**
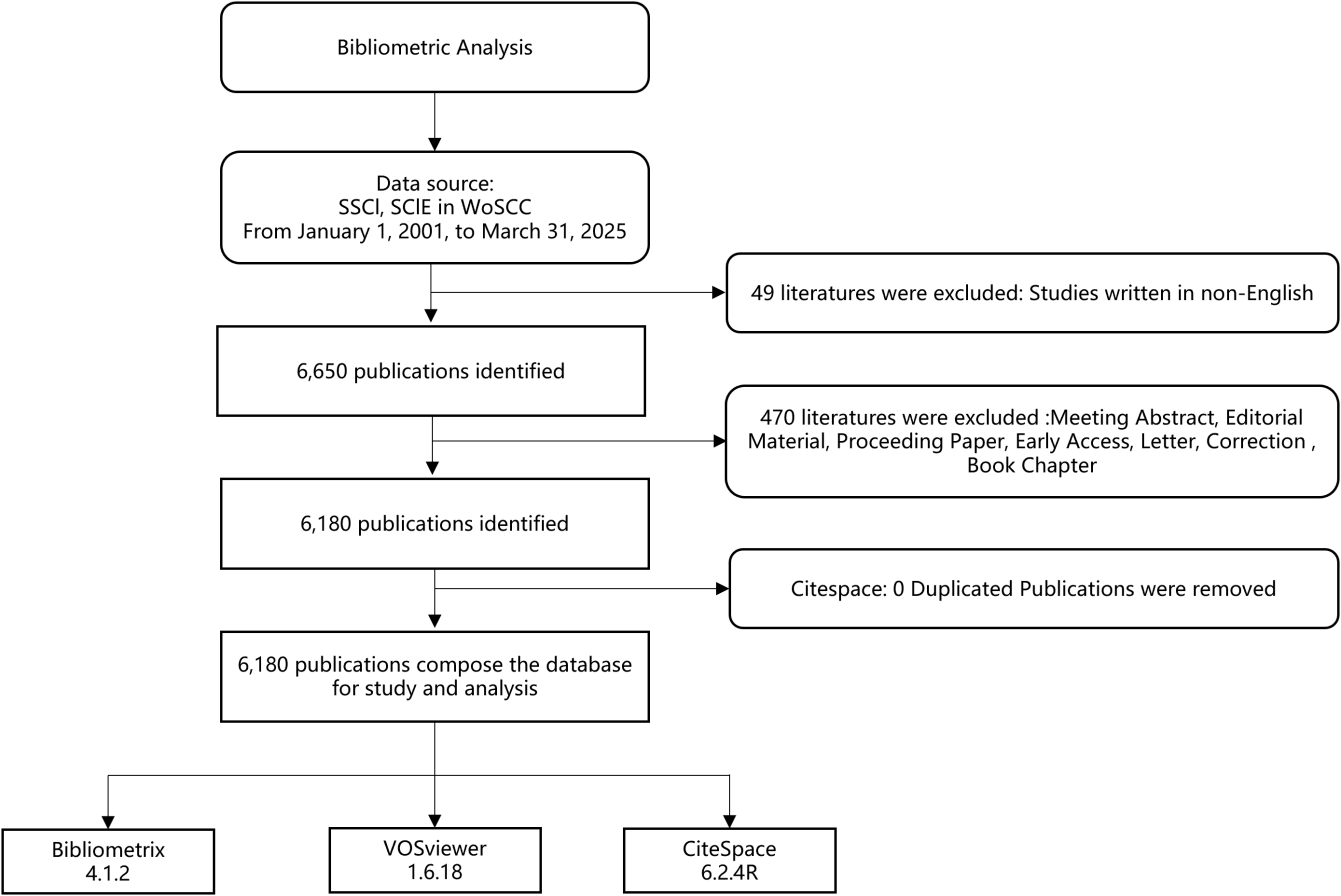
Flowchart of the screening process.

This study employed bibliometric data systematically retrieved from the Web of Science Core Collection (WoSCC), the most extensively curated citation index encompassing multidisciplinary scholarly publications. Literature search was conducted within a single day on 11 April 2025, mitigating potential biases arising from database updates. The search formula was set as follows: TS=((lung OR pulmonary OR “non-small cell” OR NSCLC OR “small cell” OR SCLC) NEAR/3 (cancer* OR carcinoma* OR tumor* OR tumour* OR neoplas* OR malignan* OR adenocarcinoma*)) AND TS=(immunotherap* OR “immune checkpoint*” OR “checkpoint inhibitor*” OR “PD-1” OR “PD1” OR “PD-L1” OR “PDL1” OR “CTLA-4” OR “CTLA4” OR “anti-PD-1” OR “anti-PD-L1” OR “anti-CTLA-4” OR “immune therap*” OR “immuno therap*”) AND TS=(“biomarker*” OR “bio-marker*”). Studies were included if they primarily investigated biomarkers in the context of lung cancer immunotherapy. The publication date was limited to the period from January 1, 2001, to March 31, 2025. Only original research articles and review articles published in English were considered. We excluded non-research items such as conference abstracts, editorials, letters, commentaries, and book chapters. Publications not relevant to the core topic and duplicate records were also excluded. The detailed screening process according to these criteria is illustrated in **Figure 1**.

### 2.2 Visualization and statistical analysis

Statistical analysis and data visualization were performed using multiple validated tools. Descriptive statistical analyses and table creation were conducted in Microsoft Excel 2021, which was also employed to generate annual global publication and citation trend graphs. For in-depth bibliometric profiling, we employed a suite of specialized software, each selected for its distinct analytical strengths to address different facets of our research questions. The Bibliometrix R-package (v4.1.2) served as the primary engine for descriptive analytics and longitudinal analysis. It was used to quantify publication trends, evaluate the productivity and impact of countries, institutions, authors, and journals, and to map the thematic evolution of the field over time. To construct and visualize the structural networks within the literature, VOSviewer (v1.6.20) was applied. This tool enabled the generation of network maps depicting collaboration among countries, institutions, and authors, as well as co-occurrence networks of keywords to identify prevailing research themes and their interconnections. For dynamic analysis aimed at detecting emerging trends and understanding the intellectual structure of the domain, CiteSpace (v6.4.R1) was utilized. Its functions included identifying references and keywords with strong citation bursts—signaling research fronts or pivotal findings—generating timeline visualizations of co-citation clusters to trace thematic development, and creating dual-map overlays of journals to reveal patterns of interdisciplinary knowledge flow. Bibliometrix provided the foundational descriptive metrics and longitudinal perspective, VOSviewer elucidated the current structural and collaborative networks within the field, and CiteSpace uncovered temporal dynamics and emerging frontiers. This integrated approach ensured that our analysis captured not only the quantity and impact of research outputs but also the intellectual structure, evolutionary pathways, and future directions of knowledge in this domain, thereby offering a comprehensive bibliometric insight. Any minor discrepancies in the results generated by different software were resolved by returning to the primary publication data and its scholarly context, ensuring the final analysis remained consistent and data-driven.

## 3 Results

### 3.1 Research profile

**Figure 2:**
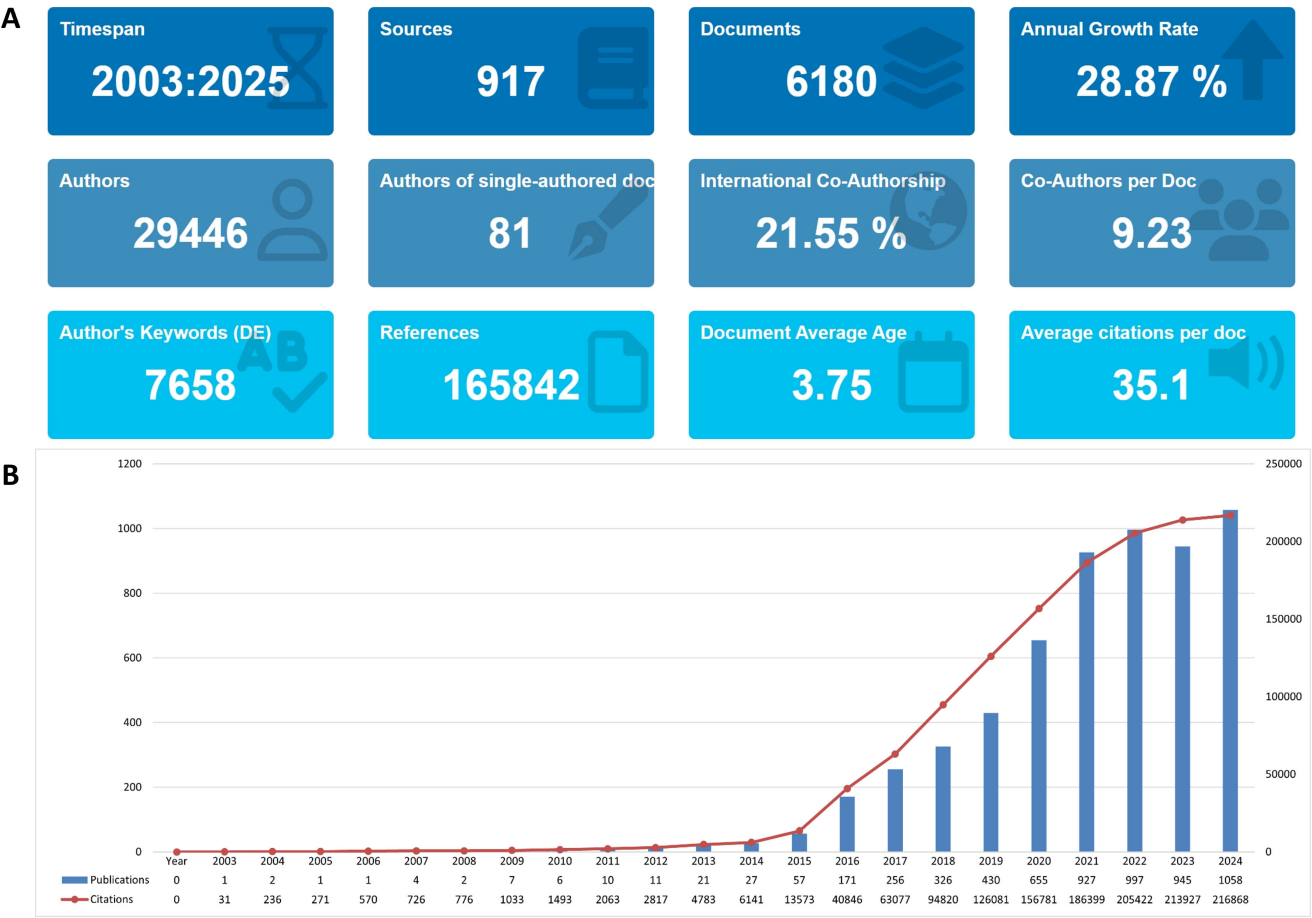
**(A)** Basic profiles of the publications included in the analysis. **(B)** Global trends in annual publication output and citation frequency related to biomarkers for lung cancer immunotherapy from 2003 to 2025.

This study systematically identified 6,180 publications on biomarkers for lung cancer immunotherapy, comprising 4,477 articles (72.44%) and 1,703 reviews (27.56%), published between January 1, 2001, and March 31, 2025. As illustrated in **Figure 2A** through R-bibliometrix analysis, the retrieved publications demonstrated substantial academic engagement with contributions from 29,446 authors, including one prolific researcher who authored 81 manuscripts. International collaborative authorship accounted for 21.55% of total publications. The analysis revealed an average of 9–10 authors per article and 7,658 unique author-defined keywords, with 165,842 cited references across all publications. Notably, the scholarly impact metrics indicated a mean citation count of 35–36 per paper and an average citation lifecycle duration of 3.75 years. These publications appeared in 917 distinct journals, showing an annual publication growth rate of 28.87%.

### 3.2 The general global trends

Analysis of the publication trajectory revealed a clear evolution from initial exploration to current maturity, characterized by three distinct developmental phases: an incubation period (2003–2014), a rapid expansion phase (2015–2022), and a recent maturation phase (2023–2025), as illustrated in **Figure 2B**. This exponential growth in scholarly output correlates with the burgeoning scientific interest and advancements in identifying predictive biomarkers for lung cancer immunotherapy, underscoring the field’s transition from exploratory research to clinical translation.

### 3.3 Analysis of countries/regions and institutions

**Figure 3:**
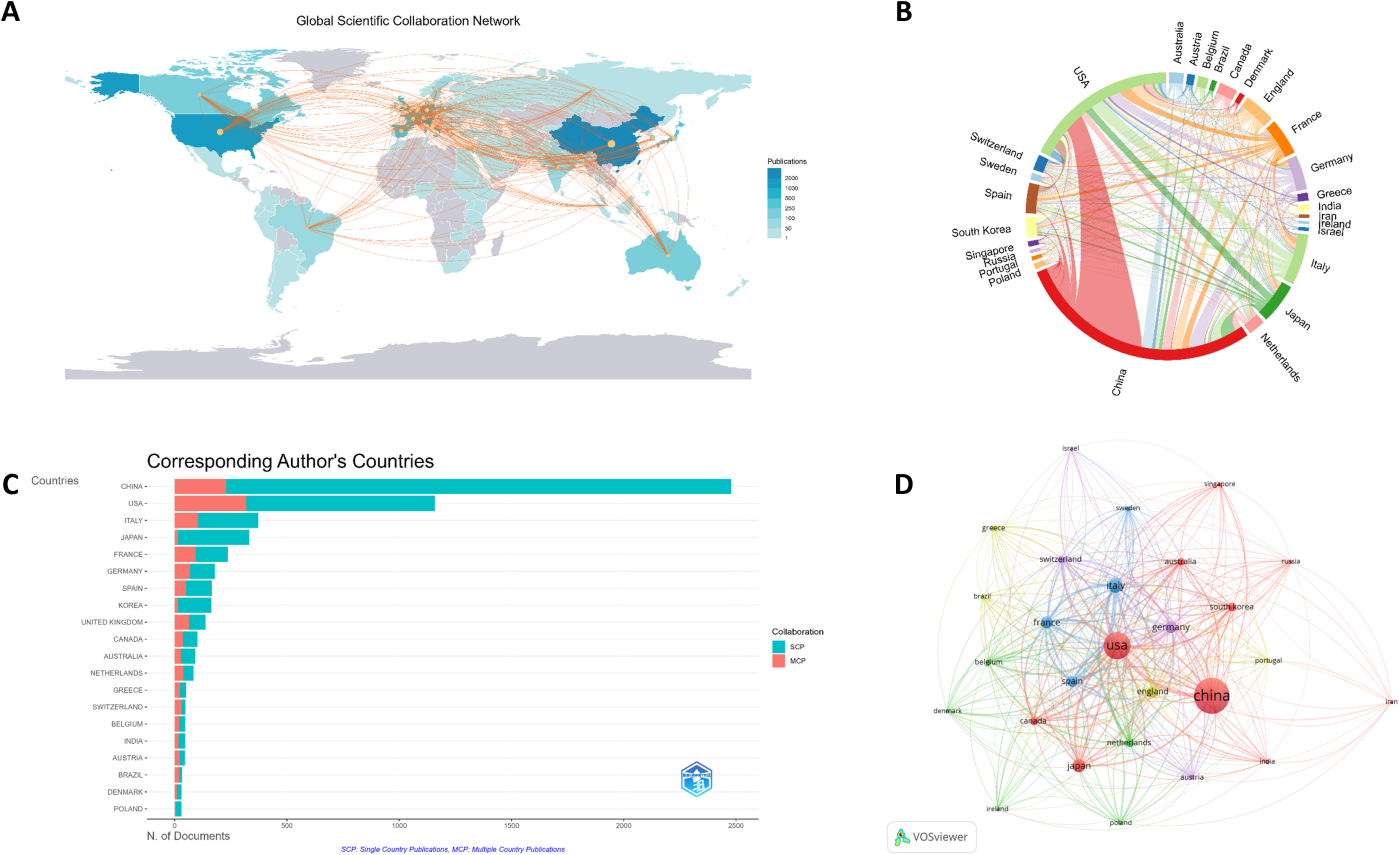
**(A)** Global distribution of scientific output and international co-authorship links. **(B)** Chord diagram of inter-country collaboration patterns. **(C)** The top 20 most productive countries, with their SCP (domestic) and MCP (international) contributions. **(D)** Network map visualizing co-authorship relationships among countries/regions.

Based on bibliometric indicators, including publication output and citation frequency, we systematically ranked the top 10 national contributions in this research field (**Table 1**). China emerged as the most productive country with 9,394 publications, followed by the United States (6,336 publications) and Italy (2,153 publications). However, when considering citation impact, the United States achieved the highest total citations (93,888 times), followed by China (41,882 times) and France (10,483 times). The H-index analysis revealed superior research quality in the United States (358), followed by China (183) demonstrating relatively high academic impact. Geospatial distribution of research outputs and international collaboration patterns were visualized using Bibliometrix (**Figure 3A**). VOSviewer analysis of international cooperation networks (**Figures 3B, D**) identified the strongest collaborative partnership between China and the United States. By analyzing both multinational collaborative publications (MCP) and single-country publications (SCP) across nations, **Figure 3C** revealed distinct geographical trends: East Asian nations, including China, Japan, and South Korea, exhibited lower MCP% values despite high productivity, while Western countries showed stronger international collaboration. This disparity suggests potential associations between research cooperation patterns and geopolitical relationships.

**Table 1:** The top 10 countries ranked by publications and citations.

Country	Publications	MCP %	H-index	Country	Citations	MCP %	H-index
CHINA	9394	9.2	183	USA	93888	27.5	358
USA	6336	27.5	358	CHINA	41882	9.2	183
ITALY	2153	28	123	FRANCE	10483	39.7	172
JAPAN	1951	4.5	95	ITALY	9855	28	123
FRANCE	1660	39.7	172	UNITED KINGDOM	8521	45.7	109
SPAIN	1476	30.1	101	GERMANY	5965	38.5	105
GERMANY	1299	38.5	105	AUSTRALIA	4787	29.7	103
KOREA	829	8.6	98	SPAIN	4893	30.1	101
UNITED KINGDOM	782	45.7	109	KOREA	4603	8.6	98
CANADA	648	36.6	76	JAPAN	8166	4.5	95

**Figure 4:**
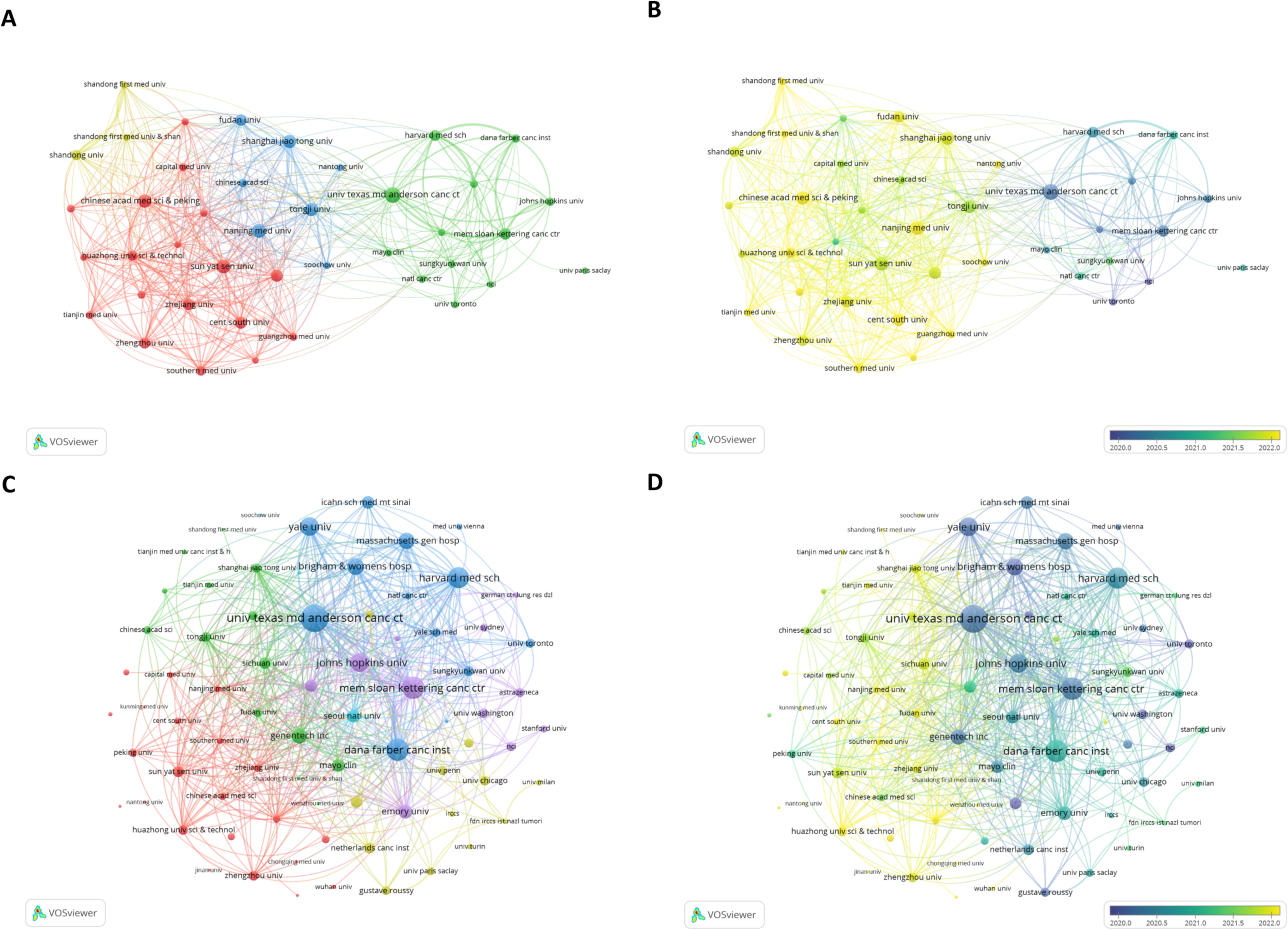
**(A)** Co-authorship network mapping the collaborative relationships among major research institutions. **(B)** Temporal evolution of the institutional collaboration network. **(B)** Citation network mapping the flow of academic influence between institutions. **(D)** Temporal overlay of the citation network.

**Table 2** presents the top 10 institutions based on publication output and total citation counts, revealing distinct geographical distributions. Among the top 10 institutions by publication volume, four of them were from the United States, four institutions were from China, and another two were from France. However, this distribution shifted significantly when ranked by total citations, with U.S. institutions constituting 70% and French institutions 30% of the top performers. Notably, Harvard University emerged as the predominant contributor, demonstrating the highest publication output (532 publications), citation impact (69,247 citations), and H-index (117), thereby establishing itself as a pioneer and leader in lung cancer immunotherapy biomarker research. The collaboration network (**Figure 4A**) and citation network (**Figure 4C**) analyses demonstrated a predominant preference for domestic collaborations over international partnerships across most institutions. International collaborations primarily occurred between elite research institutions from different nations. Temporal evolution analysis (**Figures 4B, D**) further demonstrated the escalating research activity of American and Chinese institutions in recent years, suggesting dynamic shifts in global research engagement patterns.

**Table 2:** The top 10 institutions ranked by publications and citations.

Institution	Publications	Citations	H-index	Institution	Citations	Publications	H-index
HARVARD UNIVERSITY	532	69247	117	HARVARD UNIVERSITY	69247	532	117
UNIVERSITY OF TEXAS SYSTEM	473	45270	109	UNIVERSITY OF TEXAS SYSTEM	45270	473	109
UNICANCER	461	32503	102	HARVARD UNIVERSITY MEDICAL AFFILIATES	54158	415	104
HARVARD UNIVERSITY MEDICAL AFFILIATES	415	54158	104	UNICANCER	32503	461	102
PEKING UNION MEDICAL COLLEGE	408	8839	41	UTMD ANDERSON CANCER CENTER	40453	384	101
UTMD ANDERSON CANCER CENTER	384	40453	101	INSERM	27561	367	77
INSERM	367	27561	77	MEMORIAL SLOAN KETTERING CANCER CENTER	24940	205	75
SUN YAT SEN UNIVERSITY	339	5960	39	YALE UNIVERSITY	23199	188	74
SICHUAN UNIVERSITY	274	6784	41	DANA-FARBER CANCER INSTITUTE	24606	179	71
CENTRAL SOUTH UNIVERSITY	273	5176	37	GUSTAVE ROUSSY	16766	199	71

### 3.4 Analysis of journals

**Figure 5:**
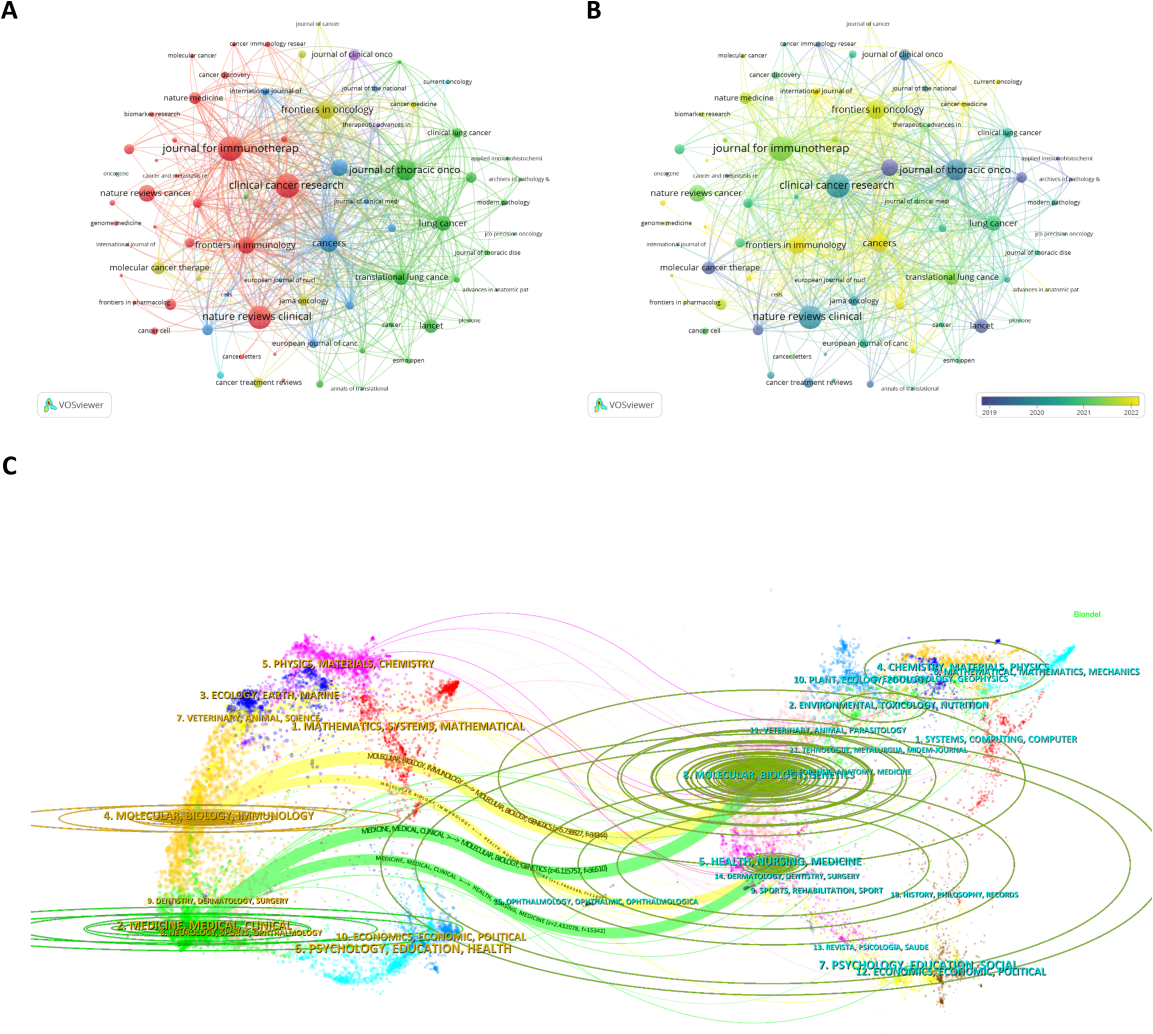
**(A)** Citation network among core journals. **(B)** Temporal evolution of the journal citation network. **(C)** Dual-map overlay of journals depicting thematic distribution and citation relationships between citing and cited journals.

We employed Bradford’s Law (**Supplementary Figure S1**) to identify 15 core journals in this research field. **Table 3** summarizes the top 10 journals ranked by publication volume and total citations, along with their impact factors and current JCR division. The most prolific journal in this domain was CANCERS (319 papers), followed by FRONTIERS IN IMMUNOLOGY (267 papers) and FRONTIERS IN ONCOLOGY (260 papers). The most cited journals were JOURNAL FOR IMMUNOTHERAPY OF CANCER (8,511 citations), CLINICAL CANCER RESEARCH (8,210 citations), and NATURE REVIEWS CLINICAL ONCOLOGY (7,806 citations), indicating their substantial influence in lung cancer immunotherapy biomarker research. **Figure 5A** presents the citation network among journals, revealing active cross-citation relationships in lung cancer immunotherapy biomarker publications. The time-overlaid network analysis in **Figure 5B** further demonstrates recent publication trends, identifying journals that have shown increasing preference for studies in this field, potentially serving as preferred publication venues for researchers.

**Table 3:** The top ten journals ranked by publications and citations.

Journal	Publications	JCR division	Journal	Citations	JCR division
CANCERS	319	Q1	JOURNAL FOR IMMUNOTHERAPY OF CANCER	8511	Q1
FRONTIERS IN IMMUNOLOGY	267	Q1	CLINICAL CANCER RESEARCH	8210	Q1
FRONTIERS IN ONCOLOGY	260	Q2	NATURE REVIEWS CLINICAL ONCOLOGY	7806	Q1
JOURNAL FOR IMMUNOTHERAPY OF CANCER	183	Q1	JOURNAL OF THORACIC ONCOLOGY	6871	Q1
TRANSLATIONAL LUNG CANCER RESEARCH	143	Q1	FRONTIERS IN ONCOLOGY	5870	Q2
LUNG CANCER	128	Q1	CANCERS	5497	Q1
SCIENTIFIC REPORTS	101	Q1	FRONTIERS IN IMMUNOLOGY	5013	Q1
CLINICAL LUNG CANCER	92	Q2	ANNALS OF ONCOLOGY	4942	Q1
INTERNATIONAL JOURNAL OF MOLECULAR SCIENCES	85	Q1	LUNG CANCER	4401	Q1
JOURNAL OF THORACIC ONCOLOGY	84	Q1	TRANSLATIONAL LUNG CANCER RESEARCH	3978	Q1

The dual-map overlay of journals generated by CiteSpace visually delineates journal thematic distributions and primary citation relationships between citing and cited journals, with colored pathways (left to right) illustrating citation trajectories. As shown in **Figure 5C**, the left-side citing journals predominantly originate from Molecular/Biology/Immunology and Medicine/Medical/Clinical domains (representing research frontiers), while the right-side cited journals cluster in Molecular/Biology/Genetics and Health/Nursing/Medicine fields (constituting knowledge foundations). Ellipse dimensions convey quantitative information: vertical axis length corresponds to publication volume, while horizontal axis reflects author count. This analytical visualization suggests that research hotspots and emerging frontiers in lung cancer immunotherapy biomarkers are progressively converging on molecular/biological/immunological mechanisms and clinical translation applications. The distinct disciplinary alignment between citing and cited journals further highlights the field’s evolving trajectory from fundamental discoveries to clinical translation applications.

### 3.5 Analysis of authors

**Figure 6:**
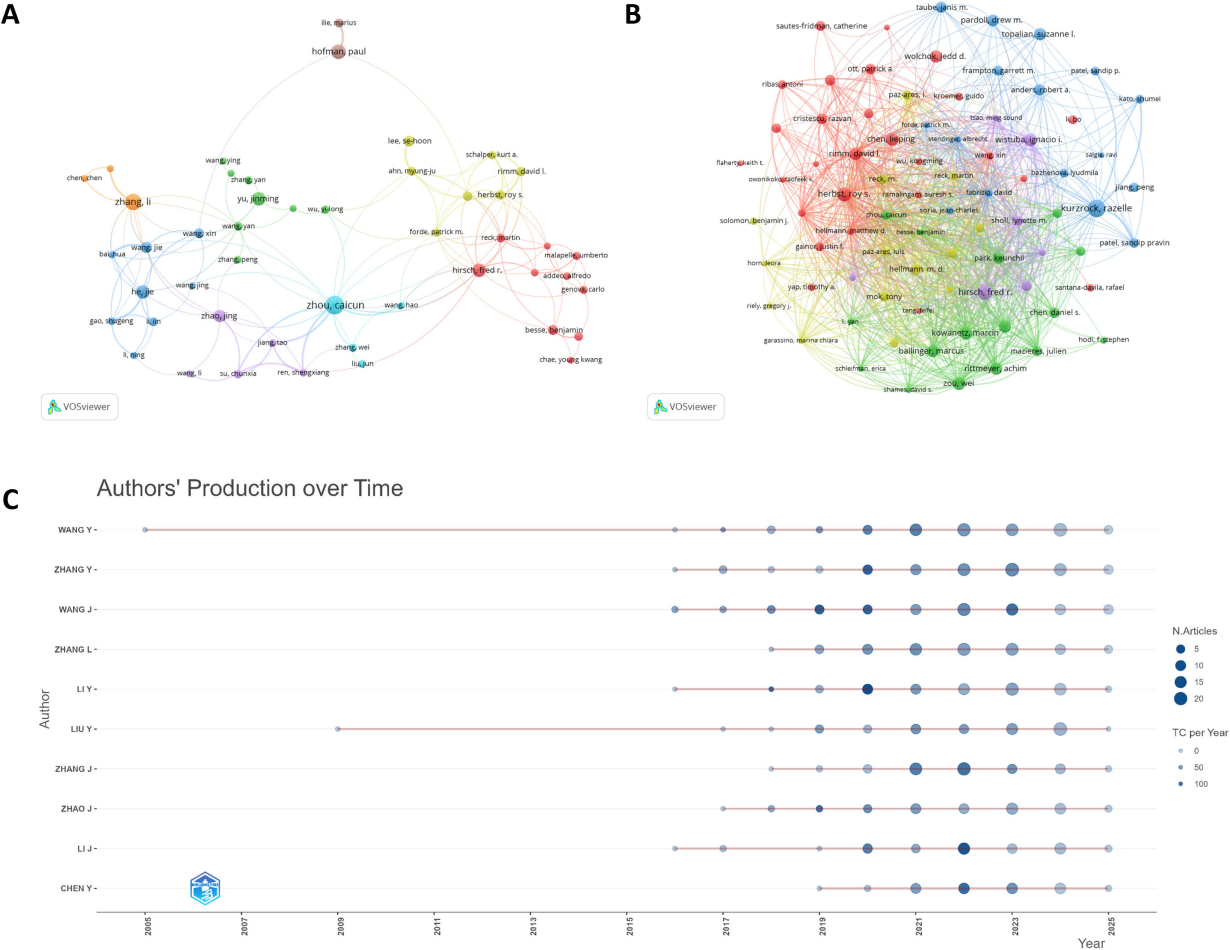
**(A)** Author collaboration network. **(B)** Author citation network. **(C)** Temporal trajectory of research productivity for the leading authors.

A total of 29,446 authors participated in research on immunotherapy biomarkers in lung cancer. Our analysis using Lotka’s Law revealed that 69.4% of authors (**Supplementary Figure S2**) contributed only one publication. **Table 4** presents the top 10 authors ranked by publication output and citation counts. WANG Y ranked first with 97 publications, followed by ZHANG Y (95 publications) and WANG J (88 publications). We visualized collaborative relationships through a co-authorship network (**Figure 6A**), where node sizes correspond to individual authors’ publication volumes, and color gradients reflect distinct collaborative clusters based on partnership intensity. In terms of scholarly impact, RAMALINGAM SS received the highest citation count (5,033 citations), followed by RECK M (4,670 citations) and HELLMANN MD (4,517 citations). The citation network analysis (**Figure 6B**) further identified author teams with strong research correlations, effectively mapping intellectual connections within the field. This network visualization not only reveals current collaboration patterns but also provides guidance for subsequent researchers in selecting potential collaborators or research directions. The size-weighting in both networks proportionally represents authors’ academic contributions, while cluster coloration demonstrates the community structure of research cooperation.

**Table 4:** The top 10 authors ranked by publications and citations.

Author	Publications	Citations	H-index	Author	Citations	Publications	H-index
WANG Y	97	2449	21	RAMALINGAM SS	5033	20	15
ZHANG Y	95	1971	21	RECK M	4670	35	18
WANG J	88	3758	26	HELLMANN MD	4517	24	21
ZHANG L	86	1405	20	KURZROCK R	4469	19	14
LI Y	78	2522	22	HIRSCH FR	4234	40	25
LIU Y	67	1249	17	PAZ-ARES L	4187	34	21
ZHANG J	66	1236	19	WISTUBA II	3898	26	18
ZHAO J	61	1918	16	WANG J	3758	88	26
LI J	59	1523	19	HERBST RS	3732	28	22
ZHOU CC	52	1866	23	ZHANG C	3142	33	16

To further investigate the top 10 most productive authors, we employed the Bibliometrix to generate a chronological visualization of their academic careers (**Figure 6C**). In this temporal graph, node size corresponds to publication output, with larger circles indicating greater productivity, while color intensity reflects annual citation counts (darker hues representing higher citations). The past decade has witnessed a notable increase in biomarker research for lung cancer immunotherapy, with growing numbers of scholars entering this field. Specifically, the timeline analysis reveals that WANG Y established an early and sustained presence in this domain, maintaining the highest cumulative publication output. Notably, although WANG J entered this research area more recently, their work demonstrates exceptional quality, as evidenced by an H-index of 26 (the highest among all analyzed authors), indicating broad recognition and impactful contributions.

### 3.6 Analysis of references and co-cited references

Highly cited publications often represent seminal works that establish foundational knowledge in a specific research domain. **Table 5** summarizes the 10 most globally cited references in the field of biomarkers for lung cancer immunotherapy. The 2016 review article “Mechanism-driven biomarkers to guide immune checkpoint blockade in cancer therapy,” published in Nature Reviews Cancer, ranks first in citation frequency. This comprehensive work emerged coinciding with the clinical approval of multiple immune checkpoint inhibitors, systematically categorizing multidimensional biomarkers and proposing a mechanism-driven development framework that continues to guide contemporary biomarker discovery. The second most cited article, “PD-L1 (B7-H1) and PD-1 Pathway Blockade for Cancer Therapy: Mechanisms, Response Biomarkers, and Combinations” (Science Translational Medicine, 2016), provides a systematic elucidation of the core mechanisms underlying PD-1/PD-L1 pathway blockade in lung cancer immunotherapy. This foundational article significantly advanced precision medicine by elucidating drug resistance mechanisms and exploring novel predictive biomarkers. Ranking third is the landmark study “Nivolumab plus Ipilimumab in Lung Cancer with a High Tumor Mutational Burden” (New England Journal of Medicine, 2018). This pivotal research prospectively validated tumor mutational burden (TMB) as a predictive biomarker for dual immunotherapy (nivolumab combined with ipilimumab), establishing crucial evidence that informed subsequent clinical practice guidelines. The study’s rigorous methodology and conclusive findings have made it a reference standard in immunotherapy biomarker research.

**Table 5:** The top 10 cited references on biomarker for immunotherapy in lung cancer.

Title	DOI	Year	Journal	First author	Global citations
Mechanism-driven biomarkers to guide immune checkpoint blockade in cancer therapy	10.1038/nrc.2016.36	2016	NATURE REVIEWS CANCER	Suzanne L Topalian	2063
PD-L1 (B7-H1) and PD-1 pathway blockade for cancer therapy: Mechanisms, response biomarkers, and combinations	10.1126/scitranslmed. aad7118	2016	Science Translational Medicine	Weiping Zou	1897
Nivolumab plus Ipilimumab in Lung Cancer with a High Tumor Mutational Burden	10.1056/NEJMoa1801946	2018	NEW ENGLAND JOURNAL OF MEDICINE	Matthew D Hellmann	1894
Atezolizumab versus docetaxel for patients with previously treated non-small-cell lung cancer (POPLAR): a multicentre, open-label, phase 2 randomized controlled trial	10.1016/S0140-6736(16)00587-0	2016	LANCET	Louis Fehrenbacher	1817
Metabolomics: beyond biomarkers and towards mechanisms	10.1038/nrm.2016.25	2016	Nature Reviews Molecular Cell Biology	Caroline H Johnson	1813
PD-L1 Expression as a Predictive Biomarker in Cancer Immunotherapy	10.1158/1535-7163.MCT-14-0983	2015	MOLECULAR CANCER THERAPEUTICS	Sandip Pravin Patel	1789
Comprehensive analyses of tumor immunity: implications for cancer immunotherapy	10.1186/s13059-016-1028-7	2016	GENOME BIOLOGY	Bo Li	1674
Tumor Mutational Burden as an Independent Predictor of Response to Immunotherapy in Diverse Cancers	10.1158/1535-7163.MCT-17-0386	2017	MOLECULAR CANCER THERAPEUTICS	Aaron M Goodman	1671
Pan-tumor genomic biomarkers for PD-1 checkpoint blockade-based immunotherapy	10.1126/science. aar3593	2018	SCIENCE	Razvan Cristescu	1634
The immune contexture in cancer prognosis and treatment	10.1038/nrclinonc.2017.101	2017	Nature Reviews Clinical Oncology	Wolf H Fridman	1550

**Figure 7:**
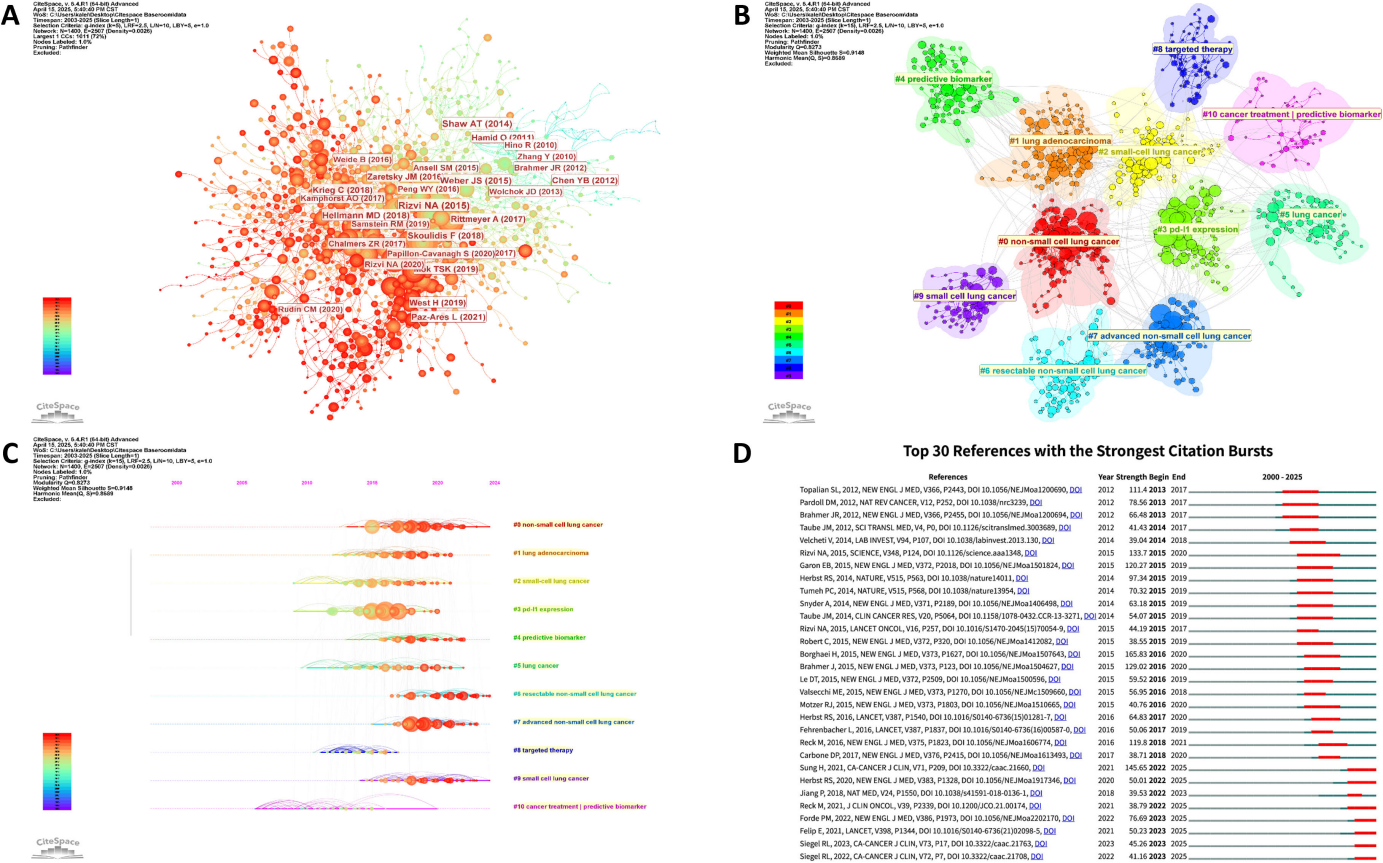
**(A)** Co-citation network of references. **(B)** Clustered co-citation network. **(C)** Timeline visualization of co-citation clusters. **(D)** Top 30 references with the strongest citation bursts from 2003 to 2025.

Co-citation network analysis, which examines the frequency of pairwise citation co-occurrences across publications, effectively reveals intellectual linkages within a research domain. Using CiteSpace, we generated a co-citation network visualization of the referenced literature (**Figure 7A**), where node size corresponds to citation counts and connecting lines indicate co-citation relationships reflecting academic connections. Cluster analysis of the co-citation network (**Figure 7B**) identified distinct research communities, while timeline visualization (**Figure 7C**) traced the temporal evolution of research focus. Predictive biomarker research was an early research focus. In the mid-term, PD-L1 research and biomarkers for immunotherapy of lung adenocarcinoma became research hotspots. Currently, research emphasis has shifted toward resectable non-small cell lung cancer (NSCLC) and advanced NSCLC, reflecting clinical demands for precision immunotherapy strategies across disease stages. This progression aligns with methodological advancements in biomarker validation and growing empirical evidence supporting personalized treatment paradigms.

Citation burst analysis, measuring sudden surges in reference citations over specific periods, serves as both an indicator of emerging scholarly attention and a marker of paradigm-shifting insights within a research field. **Figure 7D** presents the top 30 references with strongest citation bursts chronologically ordered by initiation year. The gray timeline spans 2000-2025, with red segments denoting peak citation periods (reflecting research hotspots or breakthrough discoveries) and n dark blue line indicating the citation duration. The earliest reference citation burst corresponds to the seminal NEJM study “Safety, activity, and immune correlates of anti-PD-1 antibody in cancer” (2012), which established foundational evidence for PD-L1 expression as a predictive biomarker in lung cancer immunotherapy. This landmark work directly informed the design of pivotal clinical trials (e.g., CheckMate 227, KEYNOTE-158) and enabled precision stratification in the era of personalized therapy. The longest sustained burst (5 years) was observed for the groundbreaking Science publication “Mutational landscape determines sensitivity to PD-1 blockade in non–small cell lung cancer” (2015). This study mechanistically validated tumor mutational burden (TMB) as an independent predictive biomarker while establishing methodological standards for multidimensional biomarker research. Its analytical framework continues to shape biomarker discovery pipelines in immuno-oncology.

### 3.7 Analysis of keywords

**Figure 8:**
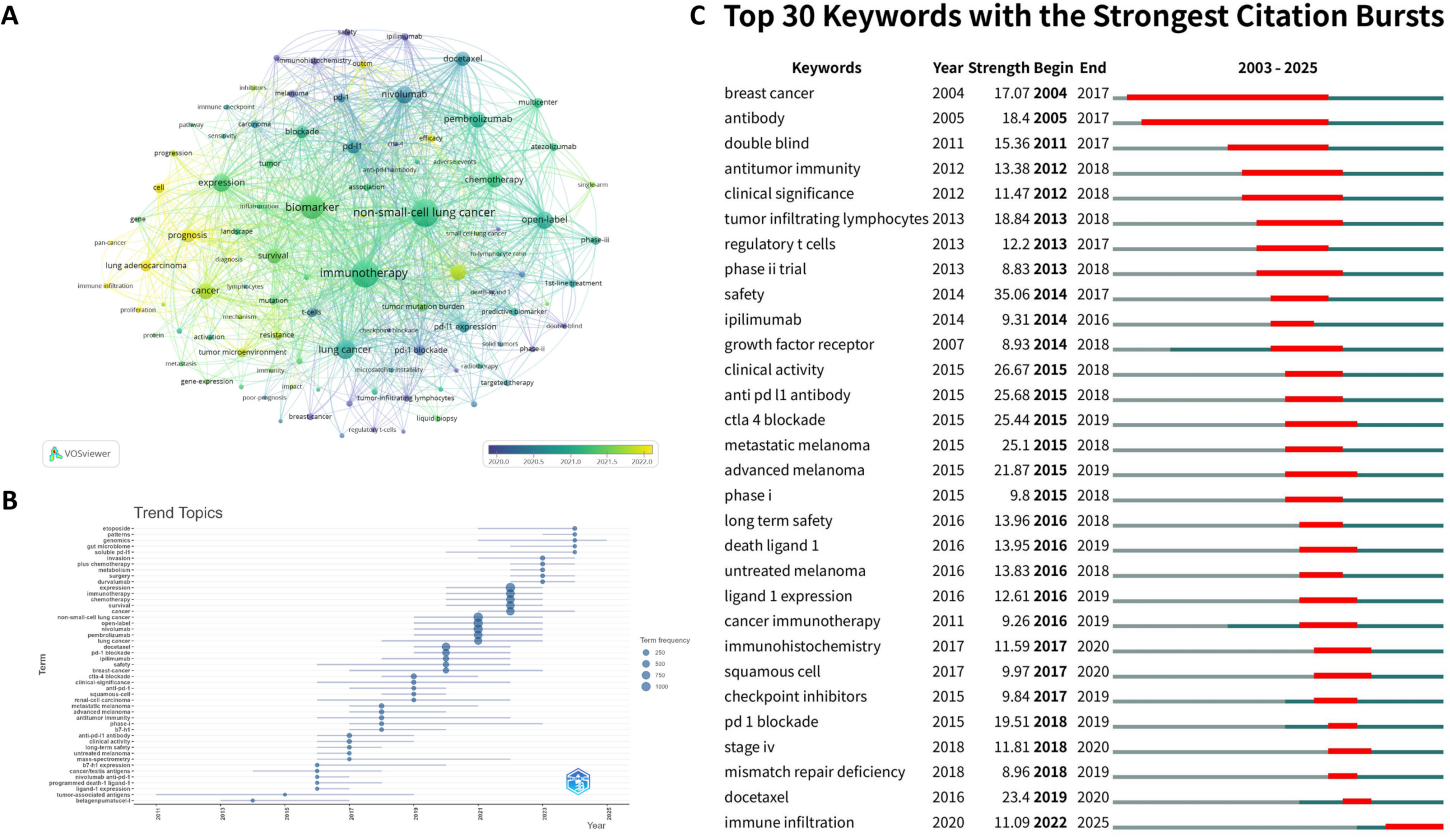
**(A)** The overlay visualization map of keyword analysis. **(B)** Evolution trends of research topics based on keyword analysis. **(C)** Top 30 keywords with the strongest citation bursts.

Keyword analysis serves as a valuable tool for mapping disciplinary knowledge structures and identifying emerging research frontiers. **Table 6** presents the 30 most frequent keywords in this domain, with “non-small-cell lung cancer” emerging as the predominant term (2,393 times), followed by “immunotherapy” (2,365 times) and “biomarker” (1,827 times)—all demonstrating strong thematic relevance. The VOSviewer-generated temporal co-occurrence network (**Figure 8A**) visually represents keyword dynamics through node size (frequency), color gradient (temporal sequence), and connection thickness (association strength). In order to more intuitively display the dynamic changes of topics within the research field, we employed R-bibliometrix to construct trend topic visualization (**Figure 8B**), where thematic trajectories are depicted as temporal spans with circular markers indicating peak activity years. The earliest emerging keyword “tumor-associated antigens” reflected researchers’ preliminary exploration of immune response mechanisms. “Belagenpumatucel-L,” as an early exploratory vaccine in the field of lung cancer immunotherapy, not only validated the feasibility of immune activation strategies in clinical trials but also promoted the deepening of biomarker research through efficacy heterogeneity. The field experienced accelerated growth post-2015, coinciding with the landmark FDA approval of PD-1 inhibitors (nivolumab) for advanced NSCLC second-line therapy. The main keywords can be divided into several categories: exploration of biomarkers for various immunotherapy drugs, such as nivolumab and pembrolizumab; exploration of the cross-cancer universality of biomarkers, such as breast cancer and melanoma; multiple predictive targets such as safety and survival; exploration of biomarkers for combination therapies, such as combined chemotherapy. Emerging keywords such as “genomics,” “gut microbiome,” and “soluble PD-L1” signal shifting research priorities toward multi-omics integration and microenvironment modulation.

**Table 6:** The top 30 co-occurrence keywords.

Rank	Keywords	Occurrences	Rank	Keywords	Occurrences
1	non-small-cell lung cancer	2393	16	blockade	571
2	immunotherapy	2365	17	pd-l1 expression	504
3	biomarker	1827	18	lung adenocarcinoma	462
4	lung cancer	1273	19	pd-1 blockade	455
5	expression	1087	20	multicenter	406
6	nivolumab	1067	21	cell	373
7	open-label	1016	22	tumor	367
8	cancer	898	23	pd-1	364
9	pembrolizumab	890	24	tumor microenvironment	351
10	chemotherapy	854	25	t-cells	339
11	immune checkpoint inhibitor	835	26	resistance	306
12	survival	786	27	phase-iii	305
13	pd-l1	746	28	tumor mutation burden	300
14	docetaxel	716	29	atezolizumab	288
15	prognosis	661	30	mutation	280

**Figure 9:**
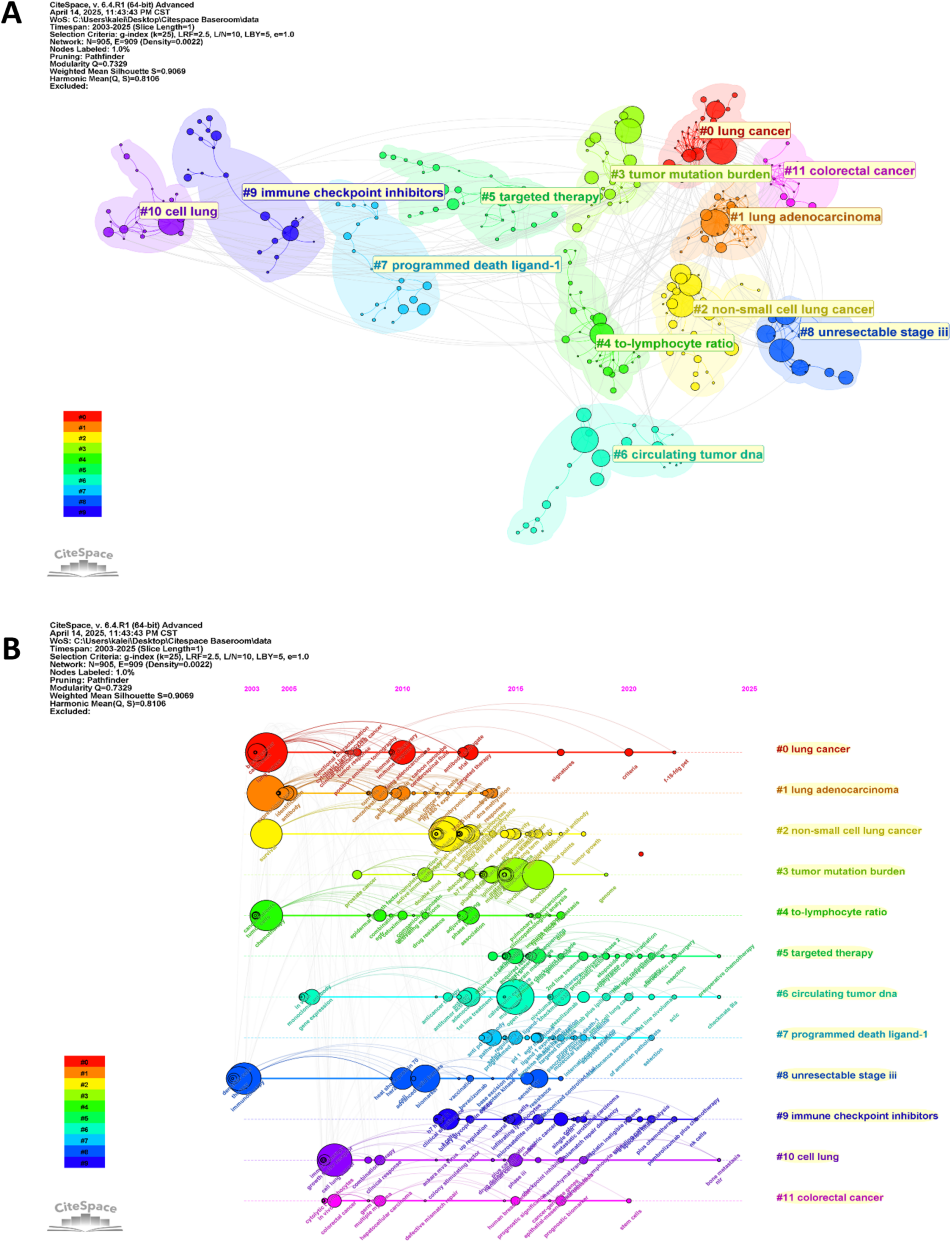
**(A)** Visualization of the keyword cluster network. **(B)** Timeline view of keyword clusters.

Furthermore, we conducted thematic clustering of high-frequency keywords using CiteSpace. The thematic clustering of keywords (**Figure 9A**) reveals a sophisticated intellectual architecture within the field. The coexistence of clusters such as #3 tumor mutation burden, #6 circulating tumor dna, and #7 programmed death ligand-1 signifies a maturation phase where traditional, single-analyte biomarkers are being systematically validated. However, the emergence and strengthening of clusters like #4 neutrophil-to-lymphocyte ratio and the persistent citation burst of immune infiltration (**Figure 8C**) indicate a paradigm shift toward a more holistic understanding of the tumor immune microenvironment (TIME). This shift is qualitative in nature, moving from a reductionist view of cancer as a driver-mutation disease to a complex ecosystem where systemic inflammation, spatial organization of immune cells, and dynamic cell-cell interactions dictate therapeutic outcomes. The interconnections between these clusters, for instance, the investigation of how TMB influences immune cell recruitment (#3 linked to immune infiltration), or how ctDNA (#6) kinetics reflect changes in the TIME, represent the most fertile ground for future discovery. This clustering analysis, therefore, does not just list topics but maps the conceptual migration of the field from static biomarker discovery to dynamic, systems-level integration. The timeline visualization (**Figure 9B**) further delineates the evolutionary trajectory of research foci within each subdomain. The terminal node in each cluster’s timeline serves as a developmental indicator, reflecting current research priorities while signaling future directions. The most recent nodes in the keyword timeline reveals a concerted shift toward context-specific integration of biomarkers. The current prominence of F-18-FDG PET within the broad #0 lung cancer cluster underscores a push to integrate radiomic signatures with molecular biomarkers. Similarly, the recent association of CheckMate 9LA with #6 circulating tumor dna, and of pembrolizumab plus chemotherapy with #9 immune checkpoint inhibitors, highlights that contemporary research is critically evaluating biomarkers within the specific contexts of advanced combination therapies and dynamic treatment response monitoring. This convergence indicates the field’s evolution from validating standalone markers toward building integrated, context-aware predictive models for clinical decision-making.

## 4 Discussion

### 4.1 Overall trajectory and phase transition of the field

This study conducted a comprehensive bibliometric analysis of 6,180 publications related to biomarkers in lung cancer immunotherapy, spanning from January 1, 2001, to March 31, 2025. By employing advanced data mining and visualization techniques, we systematically examined the current research landscape, identified emerging trends, and established a knowledge framework for this rapidly evolving field. To our knowledge, this represents the first bibliometric study specifically focused on biomarkers in lung cancer immunotherapy, providing empirically supported guidance for researchers and clinicians navigating this complex therapeutic domain. The findings offer valuable insights for optimizing biomarker discovery strategies and advancing personalized treatment approaches in immuno-oncology.

Our comprehensive analysis demonstrates a steady upward trajectory in both publication output and citation rates within the field of lung cancer immunotherapy biomarker research, reaching a plateau of high activity in recent years while maintaining sustained academic influence. The key breakthroughs in the field of lung cancer immunotherapy mainly occurred after 2015, driven by the landmark CheckMate 017/057 trials (15, 16) that first demonstrated nivolumab’s survival benefits in advanced NSCLC, coupled with the KEYNOTE-024 trial (17) establishing pembrolizumab as the first-line standard of care for PD-L1-high patients. Prior to this watershed period, immuno-oncology research predominantly focused on foundational mechanistic investigations of biomarkers, with systematic exploration of lung cancer-specific immune biomarkers remaining largely unexplored. The recent moderation in publication growth rates signals a strategic transition from exploratory discovery to rigorous validation and clinical integration of biomarkers. This progression not only reflects scientific maturation but also underscores the imperative of bibliometric analysis to map knowledge trajectories and prioritize translational research avenues.

### 4.2 Global research disparities

Our analysis reveals a notable divergence between research productivity and academic impact. While China leads in publication volume, the U.S. dominates in total citations and H-index, suggesting a higher influence per publication. This discrepancy warrants qualitative consideration. It may stem from several factors: firstly, a potential difference in the nature of research—U.S. contributions may include a higher proportion of seminal clinical trials and foundational mechanistic studies, which naturally attract more citations, whereas a larger fraction of Chinese output might be focused on validation studies or cohort analyses. Secondly, publication and collaboration strategies differ markedly. The higher MCP% (Multi-Country Publications) of the U.S. and European countries is often correlated with higher impact, as international collaborations bring diverse expertise and access to broader datasets. The relatively insular collaboration patterns observed in some high-output East Asian nations could limit the global reach and impact of their findings. This qualitative interpretation suggests that merely increasing output is insufficient; strategic international partnerships and a focus on high-risk, high-reward transformative research are critical for enhancing global academic influence. To address these disparities, we advocate breaking down academic barriers and supporting multinational cooperation, integrating data from different races and regions through multi-center cohort studies, reducing biomarker bias, which can accelerate the realization of the “precision treatment” vision and ultimately reduce the global burden of lung cancer.

### 4.3 Key journals and influential authors

We systematically evaluated journal-specific academic influence and domain maturity through quantitative analysis of core publication metrics, including article volume, citation frequency, and inter-journal citation networks. Notably, among the top 10 journals ranked by publication volume and total citations, only a minority were categorized as JCR Q2, with the majority belonging to Q1 division, reflecting both the field’s research prominence and its strong academic recognition. Temporal network analysis revealed a marked increase in annual publications for journals such as Journal for Immunotherapy of Cancer, indicating their emergence as preferred dissemination platforms for researchers in recent years. In the author analysis, WANG Y (88 publications) was identified as a pioneer who initiated research in this field earlier than counterparts and maintained the highest publication output. WANG J demonstrated substantial academic influence through the highest H-index, supported by consistently high publication counts and citation metrics. Notably, RAMALINGAM SS presented an exceptional case with relatively limited publications (n=20) yet achieved remarkable citation impact (5,033 citations). This contrast highlights RAMALINGAM SS’s exceptional focus on research quality, exemplifying a quality-over-quantity approach worthy of emulation by future investigators. The profiles of these top authors encapsulate the spectrum of impactful contributions in a translational field: sustained productivity (WANG Y), high-quality consistent output (WANG J), and paradigm-defining, highly cited clinical research (RAMALINGAM SS). The journal analysis further reveals that while specialty journals are the primary home for this research, the most influential knowledge is still disseminated through and validated by high-impact, broad-scope clinical journals. This underscores the field’s dual need for specialized forums and mainstream clinical acceptance.

### 4.4 Clinical translation focus

Co-citation and reference burst analysis delineate two predominant clinical arenas driving biomarker research: advanced/metastatic NSCLC and resectable/early-stage NSCLC. This bifurcation reflects distinct clinical goals: prolonging survival in the former and preventing recurrence in the latter, each demanding tailored biomarker strategies. Advanced NSCLC accounts for the majority of newly diagnosed lung cancer cases. Although ICIs have significantly prolonged survival, only approximately 20% of patients achieve long-term benefits (15, 16), and notable individual heterogeneity exists in treatment responses (17). This underscores the imperative for precise biomarker identification to optimize therapeutic stratification. The primary treatment goals for advanced NSCLC patients center on survival prolongation and quality-of-life improvement. Combinations of immunotherapy with chemotherapy or anti-angiogenic agents have become standard regimens (18, 19). However, differential responses to immunotherapy exist between histopathological subtypes, such as squamous cell carcinoma and adenocarcinoma, indicating the need to tailor treatment strategies by integrating driver gene mutations and tumor microenvironment characteristics. The landmark ATLANTIC trial (20) conducted the first prospective evaluation of the PD-L1 inhibitor durvalumab in EGFR-mutated/ALK-rearranged positive NSCLC, mechanistically revealing that EGFR mutations foster an immunologically inert (“immune-desert”) TME through PD-L1 downregulation and CD8+ T-cell exclusion (21). A recent study by Lin F et al. (22) published in Cell reported a “multi-cell recruitment” chimeric molecule (multi-TAC) that simultaneously activates T cells, dendritic cells (DCs), and natural killer (NK) cells, significantly enhancing therapeutic efficacy in driver mutation models. Future research should focus on integrating biomarkers with multi-omics data to guide precision stratification, aiming to optimize the selection and application of combinatorial treatment regimens for advanced NSCLC patients. Postoperative recurrence remains a principal challenge in resectable NSCLC. The efficacy of immunotherapy in reducing recurrence risk as neoadjuvant/adjuvant therapy has been validated in phase III trials (4, 23). However, heterogeneous therapeutic responses and potential immune-related adverse events necessitate refined patient stratification. Traditional biomarkers cannot meet current clinical needs due to their limitations, prompting increasing attention to emerging biomarkers with promising application potential. ctDNA clearance rate has been strongly associated with pathological complete response (pCR) and disease-free survival (DFS), as demonstrated in the CheckMate 816 trial (4). Furthermore, the MRD-EDGE platform (24) integrates whole-genome sequencing with machine learning to detect molecular residual disease (MRD) months or even years after surgery, achieving remarkable sensitivity (AUC = 0.98). These advancements underscore the critical role of multi-omics and artificial intelligence integration in recurrence surveillance. Recent mechanistic insights into tumor microenvironment (TME) dynamics further illuminate predictive biomarkers. Hu et al. (25) employed single-cell sequencing to delineate TME remodeling following neoadjuvant immunotherapy, identifying FCRL4+FCRL5+ B cells and CD16+CX3CR1+ monocytes as predictive cellular signatures. There is a need to conduct more prospective clinical trials to validate these novel biomarkers and explore stratified treatment strategies guided by multi-omics technologies for personalized management of resectable NSCLC.

The analysis of reference citation burst revealed that seven references remain in active citation burst phases. Among them, three were recent cancer statistics articles (26–28) that integrate global lung cancer epidemiology to provide a clinically oriented roadmap for biomarker research in lung cancer immunotherapy. The remaining four publications focus on clinical trials. Specifically, three correspond to the CheckMate 816 trial (4), the five-year follow-up of KEYNOTE-024 (29), and the IMpower010 trial (23), which are associated with advanced and resectable NSCLC, respectively—findings that further validate results from the co-citation analysis. The final reference, the IMpower110 trial (30), by focusing on PD-L1-selected populations, not only reinforces PD-L1 as a cornerstone biomarker for immunotherapy but also highlights the predictive value of the tumor microenvironment (TME). This suggests a need for future investigations into TME-related markers, such as CD8+ T cell density (31) and infiltration patterns of immunosuppressive cell subsets (32).

### 4.5 Emerging biomarkers and research frontiers

Keyword analysis serves as the most critical component in bibliometric studies, offering direct insights into the core themes of scientific literature while objectively revealing research frontiers and emerging trends through quantitative approaches. The trend topics visualization identifies recent high-frequency keywords including “etoposide”, “patterns”, “genomics”, “gut “microbiome”, and “soluble PD-L1”.

“genomics” emerges as the most contemporary focus, providing systematic approaches for deciphering molecular characteristics of malignancies (33), driver mutations (34), and immune microenvironment heterogeneity (35). This process provides key evidence for accurately predicting responses to immunotherapy and drug resistance mechanisms. Recent technological advancements in liquid biopsy and dynamic monitoring have significantly propelled genomic applications. Recent research by Xu et al. (36) developed blood-based Genomic Immune Subtyping (bGIS) through circulating tumor DNA analysis, effectively stratifying responders from non-responders in chemoimmunotherapy combinations. This innovation establishes a non-invasive framework for therapeutic decision-making. Zhang et al. (37) utilized single-cell sequencing to characterize the immune microenvironment after neoadjuvant immunotherapy in NSCLC. This study identified FGFBP2-positive NK cells as significantly associated with immunotherapy responses and characterized two distinct drug resistance mechanisms: Treg-enriched and insufficient immune activation types. These findings pave the way for new directions in personalized treatment. Furthermore, integrating genomics with radiomics and deep learning enables the construction of artificial intelligence-driven genomic markers. Chen M et al. (38) developed a predictive model combining CT radiomic features and gene expression data to non-invasively predict treatment response to PD-1/PD-L1 inhibitors and their risk of pneumonia. The model achieved an AUC of 0.68 for treatment response prediction and 0.64 for pneumonia risk prediction. These multimodal approaches demonstrate the potential for non-invasive treatment optimization in lung cancer immunotherapy. With continuous technological innovations and deeper mechanistic investigations, genomics is poised to play an increasingly pivotal role in lung cancer immunotherapy, driving substantial breakthroughs in precision medicine.

Etoposide, a cornerstone chemotherapeutic agent in first-line treatment for extensive-stage small cell lung cancer (ES-SCLC) (39), has garnered renewed scientific interest due to its synergistic potential with immunotherapy. Evidence has shown that etoposide-mediated activation of the STING pathway enhances chemokine secretion and promotes T-cell infiltration, thereby potentiating antitumor immune responses (40). Concurrently, etoposide-induced DNA damage repair defects (41) may serve as predictive biomarkers for immunotherapy response. Additionally, post-treatment increases in the quantity and activity of tumor-infiltrating lymphocytes (TILs), such as CD8+ T cells, correlate positively with therapeutic efficacy, positioning these parameters as potential dynamic markers of TME changes. Future studies are needed to validate the clinical utility of these markers and explore innovative strategies to overcome resistance. The prominence of “patterns” in biomarker research for lung cancer immunotherapy primarily stems from their capacity to integrate multidimensional biomarker information and systematically decode TME heterogeneity. Recent investigations demonstrate that spatial aggregation patterns of tumor-infiltrating immune cells may better reflect antitumor immune activity than conventional density measurements (42, 43). Furthermore, advanced machine learning models have successfully identified latent predictive patterns in high-dimensional datasets, enabling more accurate immunotherapy outcome predictions (44, 45). This paradigm shift reflects the evolving landscape from single-marker analysis to multidimensional dynamic assessment in lung cancer immunotherapy. In the future, integrative multi-omics pattern analysis combining genomics, radiomics, and artificial intelligence (AI) holds promise for achieving more precise personalized treatment strategies.

The increasing research focus on the “gut microbiome” as a biomarker for lung cancer immunotherapy stems from three core mechanisms. First, specific gut microbiota-derived metabolites directly activate dendritic cells (DC) and CD8+ T lymphocytes, thereby potentiating antitumor immunity (46). Second, microbial dysbiosis can induce pulmonary immune microenvironment disturbances through the gut-lung axis (47). Third, antigenic epitopes from certain gut commensals exhibit structural homology with tumor-associated antigens or autoantigens, potentially compromising immunotherapeutic efficacy via molecular mimicry (48). Recent advancements by Zhu et al. (49) demonstrated a novel predictive model integrating nine microbial metabolites identified through multi-omics analysis (metagenomics and metabolomics), achieving superior predictive performance (AUC = 0.87) compared to single-parameter biomarkers. In addition, fecal microbiota transplantation (FMT), as a microbiome-modulating intervention, has shown efficacy in enhancing immune checkpoint inhibitor responses in melanoma (50) and preclinical models (51). Currently, multiple clinical trials related to lung cancer, such as NCT05893271, are underway. The gut microbiome represents a transformative biomarker system in lung cancer immunotherapy, offering an integrated paradigm spanning predictive assessment to therapeutic intervention. With the advancement of multi-omics technologies and precision medicine, microbiome-guided therapeutic strategies are poised to become an important direction in future lung cancer immunotherapy.

The “soluble PD-L1” (sPD-L1) serves as a circulating biomarker enabling dynamic monitoring via blood samples, which is particularly suitable for advanced-stage patients or those with inaccessible tissue sampling. Mechanistic studies (52) have demonstrated that sPD-L1 directly compromises the therapeutic efficacy of ICIs by binding to PD-1 receptors and suppressing T-cell activation. Additionally, sPD-L1 levels may comprehensively reflect the interplay between the TME and systemic immunity. Liu J et al. (53) demonstrated that sPD-L1 promotes M2 macrophage polarization through activating the PI3K-AKT-mTOR pathway, enhancing interleukin-6 (IL-6) secretion by cancer-associated fibroblasts (CAFs) to form a local immunosuppressive microenvironment. Meanwhile, it is transmitted to distant organs via exosomes, inducing systemic immune suppression. Clinical validation studies have established baseline sPD-L1 levels as a prognostic indicator for overall survival (OS) in NSCLC patients undergoing immunotherapy (54). Emerging evidence suggests synergistic predictive potential when combining sPD-L1 with complementary biomarkers, including circulating tumor DNA (ctDNA) (36) and exosomal PD-L1 (55). With advancements in detection technologies and ongoing prospective clinical trials, sPD-L1 profiling is poised to become an indispensable component of precision immunotherapy strategies for lung cancer.

Keyword citation burst analysis indicates that “immune infiltration” is an intensely discussed research topic, highlighting its current importance as a key focus in biomarker research for lung cancer immunotherapy and a critical direction for future studies. Immune infiltration patterns provide critical insights into the functional status of immune cells within the TME and their dynamic interactions with malignant cells, enabling precise prediction of therapeutic response and clinical outcomes in lung cancer patients receiving immunotherapy. Clinical validation from the ORIENT-11 trials (56) demonstrated that patients with elevated PD-L1 mRNA expression and high immune scores exhibited significantly prolonged progression-free survival (PFS) and overall survival (OS) when treated with combination immunotherapy and chemotherapy. While conventional immune infiltration assessment relies on RNA sequencing or immunohistochemical techniques, recent technological advancements have substantially enhanced analytical capabilities. Notably, Chen H et al. (57) employed spatial multi-omics to characterize the immune niche in small cell lung cancer, identifying a distinct “MT2” subpopulation composed of M1-like macrophages, CD8+ T cells, and NKT cells. This immunologically active architecture showed a strong correlation with improved therapeutic response, where patients with MT2-rich tumors demonstrated extended median OS and enhanced objective response rates to PD-L1 inhibitors. Li H et al. (58) further revealed through single-cell sequencing that specific immune cell infiltration patterns in lung adenocarcinoma can promote therapeutic responses to immunotherapy. However, two critical challenges warrant attention: First, the spatial heterogeneity of tumors may lead to sampling bias in single-site biopsies, potentially misrepresenting global TME characteristics. Second, the dynamic nature of immune infiltration during disease progression and therapeutic intervention necessitates continuous monitoring. To address these limitations, developing non-invasive monitoring techniques such as liquid biopsy represents a crucial future direction for real-time tracking of immune infiltration dynamics. Furthermore, the integration of multi-omics data (genomic, metabolic, and radiomic profiles) through advanced artificial intelligence algorithms could establish comprehensive predictive models. This multidimensional approach would leverage complementary data streams to enhance prediction accuracy of immunotherapy response, ultimately facilitating efficient translation from bench research to clinical practice.

The emerging frontiers identified through our keyword analysis are not isolated trends but interconnected facets of a collective endeavor to conquer tumor heterogeneity. Qualitatively, these areas form a multi-faceted strategy: genomics offers a blueprint of tumor antigenicity; immune infiltration captures the local immune response within the tumor microenvironment; soluble PD-L1 acts as a systemic biomarker reflecting host immune status and tumor immune evasion capacity; and the gut microbiome functions as a remote modulator of systemic and anti-tumor immunity. The convergence of these themes exemplifies the current trajectory of the field. We emphasize that the future of biomarker development lies not in identifying a single stand-alone marker, but in constructing integrated, multi-dimensional models that capture the complex biology of the patient-tumor-immune system interplay.

### 4.6 Paradigm shift and future directions

This study delineates a critical yet underappreciated paradigm shift in the field: from a reductionist focus on single molecules toward a multidimensional integration of genomics, the microbiome, soluble mediators, and spatial immune architecture. This evolution, mapped through keyword and co-citation dynamics, signals a maturation toward systems-level biomarker discovery. Furthermore, our analysis uncovers a persistent geographical asymmetry: while China leads in research output, the United States and European institutions maintain superior academic influence, driven by higher rates of international collaboration and publication in high-impact journals. This disparity underscores the need for strategically fostered global partnerships to enhance translational validity. These insights offer concrete guidance for clinicians and researchers. Clinically, the rising prominence of dynamic, non-invasive biomarkers—such as soluble PD-L1, ctDNA, and gut microbiome signatures—highlights a tangible pathway toward blood-based monitoring and adaptive therapy. For future investigations, we prioritize three actionable avenues: First, establishing international consortia to validate biomarkers across diverse populations, minimizing ethnic and regional bias; second, advancing multi-omics integration coupled with AI-driven modeling to decipher tumor-immune ecosystem complexity; and third, designing prospective trials that incorporate spatially resolved biomarkers (e.g., immune infiltration patterns) to better predict responses to combination therapies.

## 5 Limitations

This study has several methodological limitations that warrant consideration. Firstly, the inherent constraints of bibliometric analytical tools restricted our data collection exclusively to the Web of Science Core Collection (WoSCC) database, which may introduce selection bias associated with database-specific coverage limitations. Secondly, our inclusion criteria focusing solely on English-language publications may have resulted in the inadvertent exclusion of impactful non-English scholarly contributions, introducing potential linguistic bias. Additionally, future studies may also consider integrating funding landscape analysis to elucidate how financial investment and national research policies shape global scientific output in this rapidly evolving field. Lastly, given the dynamic and cumulative nature of academic citation practices, recently published high-impact research could be underrepresented in the analysis, as emerging works often require extended time to accrue citation counts necessary for robust bibliometric assessment.

## 6 Conclusion

This bibliometric analysis delineates the rapid evolution and current landscape of biomarker research in lung cancer immunotherapy, revealing a paradigm shift from single-biomarker pursuit to the integrated analysis of multi-omics, systemic dynamics, and spatial microenvironment. Despite remarkable productivity, the field faces translational challenges marked by uneven global collaboration and a need for rigorous clinical validation. To realize personalized immunotherapy, future efforts must prioritize the standardization of biomarker assessment, the prospective integration of AI-enhanced multi-omics models into clinical trials, and the establishment of shared international data platforms. Concurrently, emerging frontiers including spatial transcriptomics, liquid-biopsy-based dynamic monitoring, and microbiome modulation require structured validation through globally coordinated studies. Ultimately, by fostering interdisciplinary innovation and equitable collaboration, the field can accelerate the translation of biomarker discoveries into stratified treatment strategies to improve patient outcomes worldwide.

## Data Availability

The original contributions presented in the study are included in the article/**Supplementary Material**. Further inquiries can be directed to the corresponding authors.
