# Supplementary figures and images for: Global research trends and focus on biomarkers in lung cancer immunotherapy: a comprehensive bibliometric insight and visualization analysis (2001-2025)
Source: Front Immunol. 2026 Feb 3;17:1622573. doi: 10.3389/fimmu.2026.1622573 (PMC12909568; doi:10.3389/fimmu.2026.1622573)

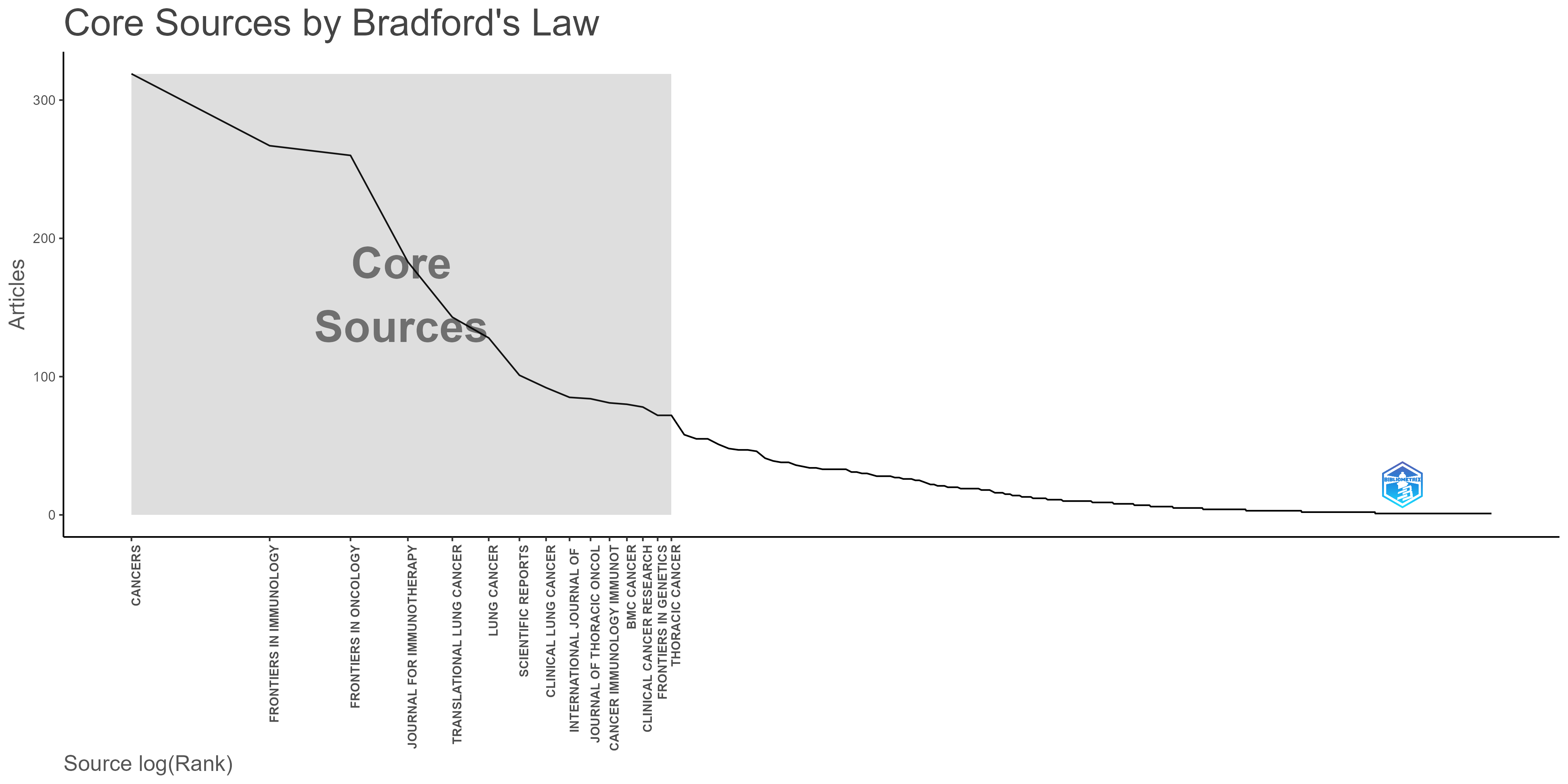

Supplement: Supplementary file 1 [file Image1.png]

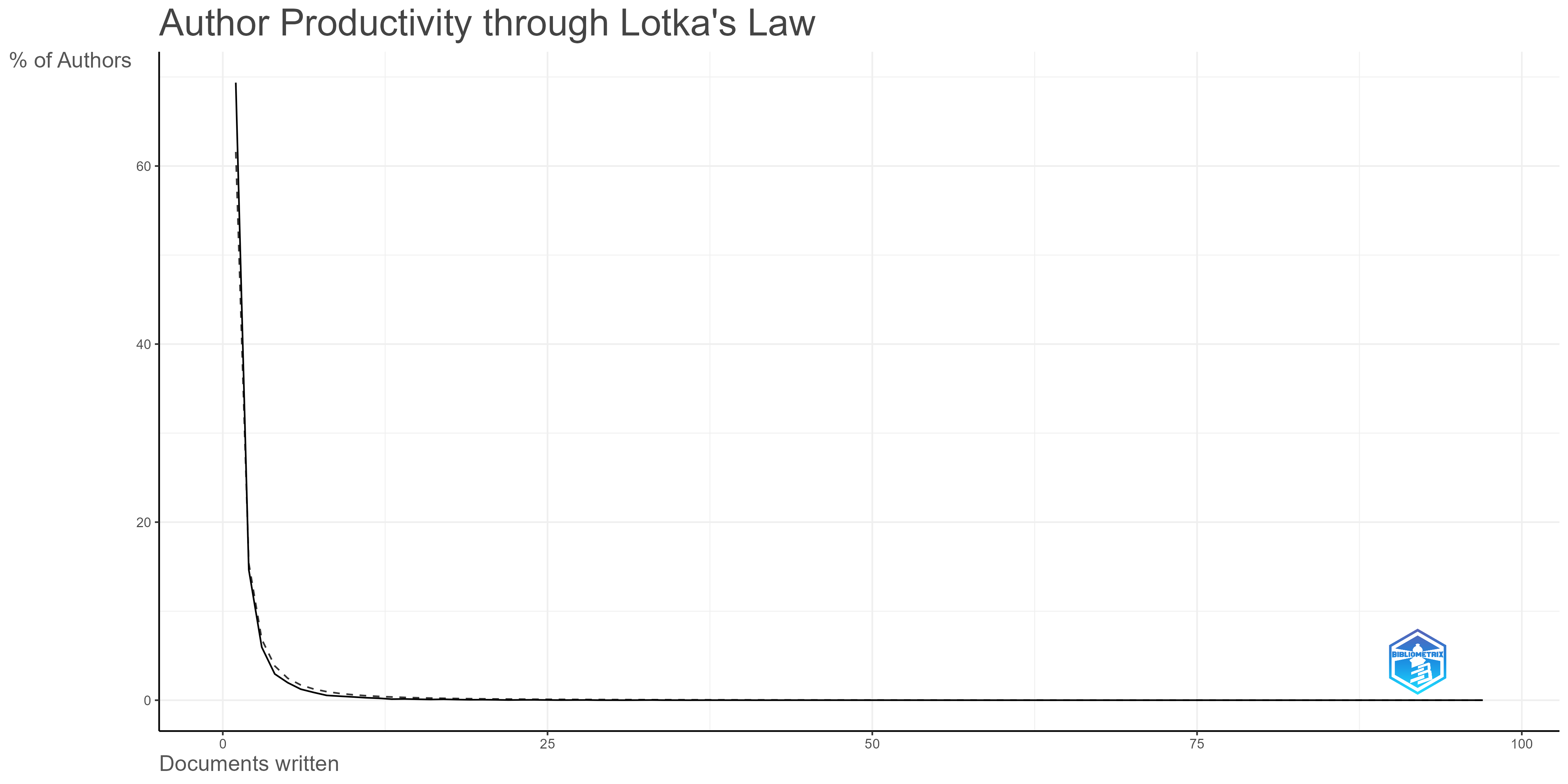

Supplement: Supplementary file 2 [file Image2.png]
